# Impact of outpatient palliative care on healthcare costs in Germany – an analysis of cancer and non-cancer patients based on health insurance data

**DOI:** 10.1186/s13561-025-00604-z

**Published:** 2025-08-20

**Authors:** Melissa Hemmerling, Sabrina Schütte, Sveja Eberhard, Katharina van Baal, Stephanie Stiel, Jona Theodor Stahmeyer

**Affiliations:** 1 Department for Health Services Research, AOK Lower Saxony, Hildesheimer Str. 273, 30519 Hannover, Germany; 2 Institute for General Practice and Palliative Care, Hannover Medical School, Carl-Neuberg-Straße 1, 30625 Hannover, Germany

**Keywords:** Palliative care, Outpatient care, Healthcare costs, Health services research, Cancer

## Abstract

**Background:**

Palliative care (PC) aims at improving the quality of life of patients suffering from life-threatening and life-limiting illnesses. International studies have found that PC is not only helpful for controlling symptoms and reducing hospital stays at the end of life, but also associated with reduced healthcare costs. However, evidence on the economic implications of outpatient PC in Germany is scarce. Accordingly, the current study aimed at assessing the impact of outpatient PC on end-of-life costs, and measuring differences between cancer and non-cancer patients who did and did not receive outpatient PC.

**Methods:**

The study involved a retrospective, cross-sectional analysis of statutory health insurance data for individuals who died in 2019 and were older than 18 years at the time of death (34,012 individuals). To explore the impact of outpatient PC on end-of-life costs, three groups were formed: (1) all individuals, (2) individuals with a cancer diagnosis and (3) individuals with no cancer diagnosis. The data were analysed descriptively and linear regression models were conducted.

**Results:**

The descriptive results showed in the group of all individuals, patients receiving outpatient PC had higher costs in all healthcare sectors compared to those who did not receive outpatient PC. Cancer patients receiving outpatient PC had higher total healthcare costs (outpatient PC: 34,822€; no outpatient PC: 26,343€; *p* < 0.001) but lower hospital costs (outpatient PC: 17,485€; no outpatient PC: 18,713€;* p* = 0,004). Non-cancer patients receiving outpatient PC had similar total healthcare costs (*p* = 0.174) but lower hospital costs (outpatient PC: 11,505€; no outpatient PC: 12,667€; *p* = 0.001).

The regression models showed significantly higher total healthcare costs (*p* < 0.001), outpatient physician costs (*p* < 0.001) and pharmaceutical costs (*p* < 0.001) for patients receiving outpatient PC in all groups. Also across all groups, hospital costs were similar between patients who were and were not receiving outpatient PC (all patients: + 40€, *p* = 0.808; cancer patients: -580€, *p* = 0.072; non-cancer patients: + 301€, *p* = 0.166).

**Conclusions:**

Unlike the findings of international studies, the present study found that outpatient PC is not associated with lower end-of-life costs. The results for hospital costs were heterogeneous, but there was a trend towards lower costs for cancer patients receiving outpatient PC. Comparability with (inter)national studies remains difficult because financing systems and access to healthcare services are not internationally consistent. Many studies recommend the early integration of PC. Further analyses should investigate the connection between the time of initiating PC and end-of-life costs.

**Trial registration:**

The main study was registered in the German Clinical Trials Register (Registration N° DRKS00024785; date of registration 6th May 2021).

**Supplementary Information:**

The online version contains supplementary material available at 10.1186/s13561-025-00604-z.

## Background

Palliative care (PC) aims at improving quality of life for both patients suffering from life-threatening and life-limiting illnesses, and their family members and informal carers [1]. Specifically, it may prevent and relieve suffering through the early identification, correct assessment and targeted treatment of pain and other physical, psychosocial and spiritual symptoms and problems. It is estimated that up to 90% of individuals require some form of PC prior to death [2–4]. Considering demographic trends, the number of individuals in need of PC is expected to continuously increase over the coming years [5].

Medical treatments in the last year of life are associated with high costs. In industrialised countries, between 8–11% (approximately 11% in Germany) of annual healthcare expenditure is spent on individuals in their last year of life (representing less than 1% of the population) [6]. Per patient, average healthcare costs in the last year of life are 22,400€, covering hospital services (55%), pharmaceuticals (19%) and outpatient care (9%). Almost 50% of end-of-life costs are attributable to the last 3 months of life [7].

Although the primary objective of PC is to improve quality of life in the last phase of life, many studies have focused on its economic aspects. Indeed, the impact of PC on resource use and healthcare costs, as well as the cost-effectiveness of specific interventions, are of major interest [8–12]. A recent review indicated that, compared to standard care, PC has the potential to lower costs at the end of life. However, the majority of the reviewed studies only focused on the impact of PC in single healthcare sectors, and the quality of the studies varied [11]. Most economic studies of PC have been conducted in the United States, Australia and Taiwan. These studies have found that cost savings associated with PC are predominantly attributable to reduced hospital (re-)admissions, shorter hospital stays and the avoidance of aggressive treatments [11].

In Germany, PC is divided into outpatient and inpatient services. Inpatient PC services provide treatment in inpatient hospices and hospital PC units. Outpatient PC services mainly include: (1) generalist outpatient PC (GPC) [13], (2) an intermediate level of PC (IPC) [14] and (3) specialist outpatient PC (SPC). GPC is the foundational PC service, and it is primarily administered by general practitioners for patients with low to medium symptom complexity [15]. The indication for specialist PC (provided by interdisciplinary teams) is greater symptom complexity and increased care needs [16]. Finally, the intermediate level of PC aims at providing care to patients whose needs cannot be met by GPC, but who do not yet require SPC [14]. In addition, patients with oncological diseases also have access to special oncological PC services (OS), provided by oncologists [17].

Evidence on the economic implications of outpatient PC in Germany is scarce. Gaertner et al. (2013) analysed the effect of inpatient PC on healthcare costs for cancer patients in the last 6 months of life [18]. Based on data from a nationwide health insurance company, a retrospective matched-pair analysis showed that total healthcare costs (associated with hospital services, pharmaceuticals, additional medical services and medical aids) were significantly higher in the group of cancer patients receiving inpatient PC, compared to a control group. Other national studies analysing the economic implications of outpatient PC have only focused on the cost difference between generalist and specialist outpatient PC [19] or assessed the costs of specialist outpatient PC from the perspective of statutory health insurance [20].

To the best of our knowledge, only a few studies have explored the economic implications of outpatient PC in Germany.

### Study aim

The current analysis aimed at: (1) assessing the impact of outpatient PC on healthcare expenditure in the last year of life and (2) assessing differences in end-of-life costs between cancer and non-cancer patients who were or were not receiving outpatient PC.

## Methods

### Study design

Statutory health insurance (SHI) data from ‘Allgemeine Ortskrankenkasse’ Lower Saxony (AOKN) was used to conduct a retrospective cross-sectional study to assess the impact of the provision of outpatient PC on end-of-life costs. With approximately three million insured members and a market share of 37%, AOKN is the largest SHI in Lower Saxony (one of 16 federal states in Germany) [21]. SHI companies collect sociodemographic data, information on diagnoses based on the *International Statistical Classification of Diseases and Related Health Problems* (10th Revision) (ICD-10) and healthcare utilisation, for billing purposes.

### Study population

**Fig. 1:**
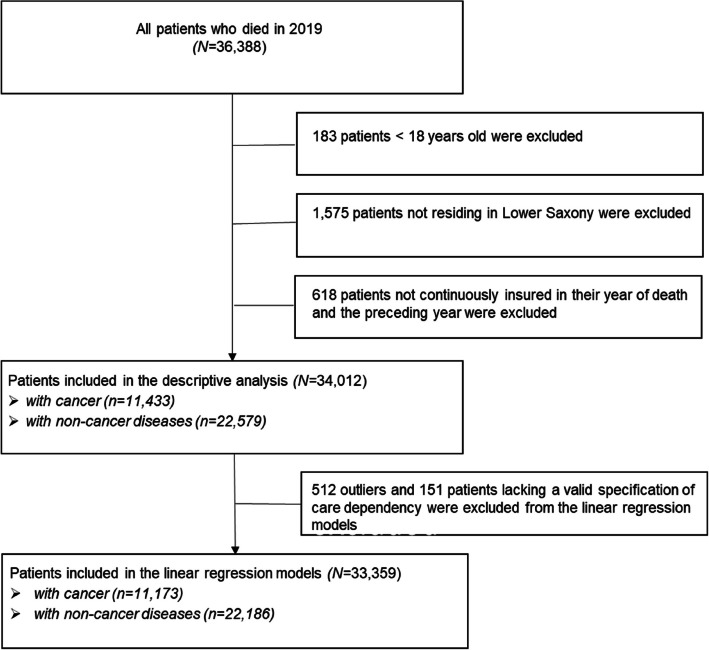
Flowchart of the analysed patients

Data for all patients who died in 2019 and were older than 18 years at the time of death were included in the analyses. We excluded minors for data protection reasons because the number of cases is very small and in Germany PC for minors is organized differently compared to PC for adults. Additionally, only individuals residing in Lower Saxony who were continuously insured in their year of death and the preceding year were included. In total, data for 34,012 individuals were considered in the analysis. To assess differences in end-of-life costs and the economic implications of receiving outpatient PC between cancer and non-cancer patients, three groups were formed, comprised of: (1) all individuals (2) individuals with a cancer diagnosis and (3) individuals with no cancer diagnosis (Fig. 1).

### Data processing

Initially, data were sorted according to whether patients received outpatient PC in their last year of life. The different forms of outpatient PC (i.e. GPC, IPC, SPC, OS) were identified by special billing codes. The data were also analysed for patients’ age at death, sex, chronic and potentially life-limiting diseases, and common (i.e. widespread) diseases. Table 1 displays patients’ diseases, classified according to ICD-10 codes.

**Table 1:** Included diseases

**Diseases**	**ICD-10 codes**
Dementia	F00, F01, F03, G30
Diabetes	E10–E14
Hypertension	I10–I15
Coronary heart disease	I25
Heart failure	I50
Asthma	J45
Chronic obstructive pulmonary disease (COPD)	J44
Depressive disorder	F32, F33
Renal failure	N17–N19
Cancer	C00–C96, without C44
Solid cancer	C00–C76 and C80, without C44
Lymphoid/haematopoietic neoplasms cancer	C81–C96
Metastatic cancer	C77, C78, C79
Parkinson’s	G20
Myocardial infarction	I21, I22
Stroke	I60–I64

The subgroups cancer patients and non-cancer patients were identified by having or not having a cancer diagnosis. Patients were classified as suffering from a disease if their specific diagnosis was coded in at least two different quarters in the outpatient sector or at least once during an inpatient hospital stay (main or secondary diagnosis) in their last year of life [22]. Finally, healthcare costs referred to the following sectors: (1) outpatient physician services (including GPC, IPC and OS), (2) pharmaceuticals, (3) hospital services, (4) SPC and (5) inpatient hospice services. All documented healthcare costs in the last year of life (i.e. 365 days before death) were considered.

### Statistical analysis

Initially, the data were analysed descriptively using the IBM Statistical Package for Social Sciences software, version 25 (SPSS Inc., Chicago, IL/USA). Differences in patient characteristics and costs between subgroups (e.g. receiving vs. not receiving outpatient PC, cancer vs. non-cancer diagnosis) were analysed using a *t*-test or chi-square test, depending on the type of variable. In order to adjust for differences in sex and age as well as morbidity, which might impact end of life costs in individuals receiving or not receiving outpatient PC, we performed linear regression models (ordinary least squares). The models were conducted for total healthcare costs, hospital costs, pharmaceutical costs and outpatient physician costs. The analysis controlled for age (age groups: under 50 years, 50–69 years, 70–79 years, 80–89 years, older than 89 years), sex, care dependency and morbidity. Categorical variables with a number of variations were dummy coded. Outlier cases were identified and excluded, based on standardized residues, using three standard deviations. Statistical significance was indicated by *p* < 0.05. The analysed data contained no direct personal references.

### Ethics and data protection

The study comprised part of the multi-perspective observational study “Analysing the administration of an intermediate level of outpatient PC in Germany and developing recommendations for improvement” (Polite), funded by the Innovation Fund of the German Federal Joint Committee (Grant N° 01VSF20028) [23]. The study was registered in the German Clinical Trials Register (Registration N° DRKS00024785; date of registration 6th May 2021) and is searchable under the International Clinical Trials Registry Platform Search Portal of the World Health Organization under the German Clinical Trials Register number.

## Results

### Patients’ sociodemographic variables, morbidity and costs

Table 2 displays patients’ mean age, sex distribution, care degree and morbidity.

**Table 2:** Patients’ sociodemographic characteristics and morbidity

		**All patients**				**Patients with cancer**				**Patients with non-cancer diseases**			
		Outpatient PC				Outpatient PC				Outpatient PC			
		Yes 31.5% (*n* = 10,722)	No 68.5% (*n* = 23,290)	Total (*N* = 34,012)	***p***	Yes 52.2% (*n* = 5,972)	No 47.8% (*n* = 5,461)	Total (*n* = 11,433)	***p***	Yes 21.0% (*n* = 4,750)	No 79.0% (*n* = 17,829)	Total (*n* = 22,579)	***p***
**Age at death (years)**	Average value	80.1	79.2	79.5	< 0.001*	75.9	78.6	77.2	< 0.001*	85.5	79.3	80.6	< 0.001*
	SD ±	12.0	13.1	12.8		12.2	11.4	12.0		9.3	13.6	13.1	
**Sex**	Female	56.4%	51.2%	52.8%	< 0.001*	48.1%	42.9%	45.6%	< 0.001*	66.8%	53.8%	56.5%	< 0.001*
	Male	43.6%	48.8%	47.2%		51.9%	57.1%	54.4%		33.2%	46.2%	43.5%	
**Care degree **^**a**^	No care degree	3.5%	26.5%	19.2%	< 0.001*	5.2%	24.9%	14.6%	< 0.001*	1.3%	27.0%	21.6%	< 0.001*
	1	0.5%	1.9%	1.4%		0.7%	2.0%	1.3%		0.3%	1.8%	1.5%	
	2	10.7%	16.9%	15.0%		18.3%	22.3%	17.8%		6.9%	15.3%	13.5%	
	3	19.1%	19.1%	19.1%		21.1%	21.0%	21.1%		16.7%	18.5%	18.1%	
	4	36.7%	20.7%	25.8%		36.7%	19.4%	28.5%		36.7%	21.1%	24.4%	
	5	29.4%	14.9%	19.5%		22.6%	10.4%	16.8%		38.0%	16.2%	20.8%	
**Morbidity **^**b**^	Dementia	36.7%	33.3%	34.4%	< 0.001*	19.8%	24.0%	21.8%	< 0.001*	57.9%	36.2%	40.8%	< 0.001*
	Diabetes	38.4%	39.6%	39.2%	**0.031**	37.3%	39.5%	38.4%	0.015*	39.7%	39.6%	39.7%	**0.917**
	Hypertension	84.7%	83.0%	83.5%	< 0.001*	81.5%	85.4%	83.4%	< 0.001*	88.7%	82.3%	83.6%	< 0.001*
	Coronary heart disease	33.6%	37.7%	36.4%	< 0.001*	29.6%	38.1%	33.65	< 0.001*	38.7%	37.6%	37.8%	**0.159**
	Heart failure	45.6%	50.1%	48.7%	< 0.001*	36.7%	48.5%	42.4%	< 0.001*	56.7%	50.6%	51.9%	< 0.001*
	Asthma	6.6%	6.5%	6.5%	**0.707**	7.4%	7.1%	7.2%	**0.514**	5.7%	6.3%	6.2%	**0.084**
	COPD	24.7%	24.2%	24.4%	**0.339**	26.8%	28.4%	27.6%	**0.057**	22.0%	22.9%	22.7%	**0.168**
	Depressive disorder	30.4%	24.8%	26.5%	< 0.001*	28.4%	24.8%	26.7%	< 0.001*	32.9%	24.7%	26.4%	< 0.001*
	Renal failure	50.7%	51.3%	51.1%	**0.307**	47.2%	56.3%	51.5%	< 0.001*	55.2%	49.8%	50.9%	< 0.001*
	Cancer	55.7%	23.4%	33.6%	< 0.001*	100.0%	100.0%	100.0%	**-**	0.0%	0.0%	0.0%	**-**
	Solid tumors	52.6%	21.2%	31.1%	< 0.001*	94.4%	90.2%	92.4%	< 0.001*	0.0%	0.0%	0.0%	**-**
	Metastatic cancer	36.0%	8.0%	16.8%	< 0.001*	64.6%	34.1%	50.0%	< 0.001*	0.0%	0.0%	0.0%	**-**
	Lymphoid/haemato- poietic cancer	5.1%	3.0%	3.7%	< 0.001*	9.1%	12.9%	10.9%	< 0.001*	0.0%	0.0%	0.0%	**-**
	Parkinson’s	5.9%	5.1%	5.3%	0.002*	3.1%	4.0%	3.6%	0.008*	9.3%	5.4%	6.2%	< 0.001*
	Myocardial infarction	2.7%	6.7%	5.5%	< 0.001*	1.9%	5.3%	3.6%	< 0.001*	3.7%	7.2%	6.4%	< 0.001*
	Stroke	6.0%	8.5%	7.7%	< 0.001*	4.6%	7.6%	6.1%	< 0.001*	7.6%	8.8%	8.6%	0.010*

*PC* palliative care, *SD* standard deviation, *COPD* chronic obstructive pulmonary disease

^a^In Germany, individuals who are limited in their independence and everyday competence receive a degree of care rating, ranging from 0–5. The rating indicates the severity of the impairment, with higher ratings indicating greater limitation in everyday life (*n* = 141 (0.4%) patients lacked a care dependency rating)

^b^ICD Codes: dementia (F00, F01, F03, G30), diabetes (E10–E14), hypertension (I10–I15), coronary heart disease (I25), heart failure (I50), asthma (J45), COPD (J44), depressive disorder (F32, F33), renal failure (N17–N19), solid tumors (C00–C76 and C80, without C44), metastatic cancer (C77, C78, C79), lymphoid/haematopoietic cancer (C81–C96), Parkinson’s (G20), myocardial infarction (I21, I22), stroke (I60–I64)

#### All patients (*N* = 34,012)

The mean age of the cohort at the time of death was 79.5 years (*SD*: 12.8), and the gender distribution was 52.8% women and 47.2% men (Table 2). The three most frequent illnesses were hypertension (83.5%), renal failure (51.1%) and heart failure (48.7%). One-third of the cohort (31.5%) received outpatient PC (any type) (Table 2). The proportion of women was higher among those receiving outpatient PC than among those who did not receive outpatient PC (outpatient PC: 56.4%; no outpatient PC: 51.2%; *p* < 0.001). Additionally, patients receiving outpatient PC were significantly older at the time of death (80.1 vs. 79.2 years, *p* < 0.001). Cancer was significantly more frequent in the group receiving outpatient PC (55.7% vs. 23.4%; *p* < 0.001).

Mean healthcare costs (per patient) in the last year of life for the total sample were 21,229€ (Table 3). Most of these costs were attributable to hospital services (14,321€), while lesser amounts were attributable to pharmaceuticals (4,372€), outpatient physician services (1,925€), inpatient hospice services (189€) and SPC (422€). Patients receiving outpatient PC had significantly higher mean total healthcare costs than those who did not receive these services (26,838€ vs. 18,648€, *p* < 0.001) (Table 3). Furthermore, patients receiving outpatient PC recorded higher mean costs in all other sectors.

**Table 3:** Costs in the last year of life, per patient

**Costs (in €)**		All patients				Patients with cancer				Patients with non- cancer diseases			
		Outpatient PC				Outpatient PC				Outpatient PC			
		Yes (*n* = 10,722)	No (*n* = 23,290)	Total (*N* = 34,012)	***p***	Yes (*n* = 5,972)	No (*n* = 5,461)	Total (*n* = 11,433)	***p***	Yes (*n* = 4,750)	No (*n* = 17,829)	Total (*n* = 22,579)	***p***
Total healthcare costs	Average value	26,838	18,648	21,229	< 0.001*	34,822	26,343	30,772	< 0.001*	16,800	16,291	16,398	**0.174**
	SD	26,939	27,932	27,883		27,915	30,836	29,649		21,856	26,538	25,624	
	CI	26,328 – 27,348	18,289 – 19,006	20,933 – 21,526		34,114 – 35,530	25,525 – 27,161	30,228 – 31,315		16,178 – 17,421	15,901 – 16,680	16,063 – 16,732	
Hospital costs	Average value	14,836	14,084	14,321	0.003*	17,485	18,713	18,071	0.004*	11,505	12,667	12,422	0.001*
	SD	19,294	25,201	23,502		18,655	26,347	22,665		19,567	24,667	23,690	
	CI	14,471 – 15,201	13,761 – 14,408	14,071 – 14,571		17,012 -17,958	18,014 – 19,412	17,656 – 18,487		10,948 – 12,062	12,305 – 13,029	12,113 – 12,731	
Outpatient physician costs	Average value	2,529	1,647	1,925	< 0.001*	3,049	2,222	2,654	< 0.001*	1,875	1,471	1,556	< 0.001*
	SD	3,282	3,685	3,586		3,291	3,862	3,598		3,152	3,611	3,523	
	CI	2,467 – 2,591	1,600 – 1,694	1,887 – 1,963		2,965 – 3,132	2,119 – 2,324	2,588 – 2,720		1,785 – 1,965	1,418 – 1,524	1,510 – 1,602	
Pharmaceutical costs	Average value	7,537	2,915	4,372	< 0.001*	11,255	5,403	8,460	< 0.001*	2,863	2,153	2,302	< 0.001*
	SD	14,032	7,719	10,367		17,323	12,717	15,573		5,291	5,083	5,135	
	CI	7,271 – 7,803	2,816 – 3,014	4,262 – 4,482		10,815 – 11,694	5,066 – 5,741	8,174 – 8,745		2,713 – 3,014	2,078 – 2,227	2,235 – 2,369	
Hospice costs	Average value	597	1	189	< 0.001*	983	5	516	< 0.001*	112	0	24	< 0.001*
	SD	3,982	69	2,253		4,931	133	3,598		2,190	29	1,006	
	CI	522 - 672	1 - 2	165 - 213		858 – 1,108	1 - 8	450 - 582		50 - 174	0 - 1	11 - 37	
SPC	Average value	1,339	-	422	**-**	2,050	-	1,071	**-**	445	-	94	**-**
	SD	3,549	-	2,088		4,239	-	3,230		2,101	-	980	
	CI	1,272 – 1,406	-	400 - 444		1,943 – 2,158	-	1,012 – 1,130		385 - 504	-	81 - 106	

*PC* palliative care, *SD* standard deviation, *SPC* specialist palliative care, *CI* 95% confidence interval

#### Patients with cancer (*n* = 11,433)

Among the patients with at least one cancer diagnosis, the gender distribution was 45.6% female and 54.4% male. The mean age at the time of death was 77.2 years (*SD*: 12.0) (Table 2). More than half of the patients in this group (52.2%) received some form of outpatient PC. Patients receiving outpatient PC suffered more frequently from metastatic cancer (64.6% vs. 34.1%, *p* < 0.001). The proportion of women was higher among the group of patients receiving outpatient PC than the group of patients who did not receive outpatient PC (48.1% vs. 42.9%, *p* < 0.001). Additionally, patients receiving outpatient PC tended to die at a younger age than patients who did not receive outpatient PC (75.9 vs. 78.6, *p* < 0.001). Aside from asthma and depression, the considered non-cancer diseases were observed less frequently among patients receiving outpatient PC.

Mean healthcare costs in the last year of life were 30,772€ (Table 3). Most of these costs were attributable to hospital services (18,071€), while lesser amounts were attributable to pharmaceuticals (8,460€), outpatient physician services (2,654€), inpatient hospice services (516€) and SPC (1,071€). Patients receiving outpatient PC had higher mean healthcare costs than patients who did not receive these services (34,822€ vs. 26,343€, *p* < 0.001) (Table 3). In particular, pharmaceutical costs were higher for patients receiving outpatient PC (11,255€ vs. 5,403€, *p* < 0.001). Mean outpatient physician costs (3,049€ vs. 2,222€, *p* < 0.001) and inpatient hospice costs (983€ vs. 5€, *p* < 0.001) were also higher among patients receiving outpatient PC. Patients receiving outpatient PC documented lower mean hospital costs (17,485€ vs. 18,713€, *p* = 0.004).

#### Patients with non-cancer diseases (*n* = 22,579)

For patients with non-cancer diseases, the mean age of death was 80.6 years (*SD*: 13.1), and the gender distribution was 56.5% women (Table 2). In this group, the three most common illnesses were hypertension (83.6%), heart failure (51.9%) and renal failure (50.9%). In total, 21.0% received (any form of) outpatient PC (Table 2). On average, patients receiving outpatient PC were older than those who did not receive outpatient PC (85.5 vs. 79.3 years, *p* < 0.001). The proportion of women was higher among those receiving outpatient PC than among those who did not receive outpatient PC (66.8% vs. 53.8%, *p* < 0.001). Relative to those who did not receive outpatient PC, those receiving outpatient PC more frequently suffered from dementia (57.9% vs. 36.2%, *p* < 0.001). In contrast, those who did not receive outpatient PC more frequently suffered from a myocardial infarction (7.2% vs. 3.7%, *p* < 0.001) or stroke (8.8% vs. 7.6%, *p* = 0.010).

Mean healthcare costs in the last year of life were 16,398€ (Table 3). Most of these costs were attributable to the hospital sector (12,422€), while smaller amounts were attributable to pharmaceuticals (2,302€), outpatient physician services (1,556€), hospice services (24€), and SPC (94€). No significant difference in mean total healthcare costs emerged between subgroups (16,800€ for those receiving outpatient PC vs. 16,291€ for those who did not receive outpatient PC, *p* = 0.174). However, those receiving outpatient PC registered lower mean hospital costs (11,505€ vs. 12,667€, *p* = 0.001) and higher costs related to outpatient physician services (1,875€ vs. 1,471€, *p* < 0.001) and pharmaceuticals (2,862€ vs. 2,153€, *p* < 0.001).

### Results of the linear regressions using cost variables: outpatient palliative care and costs in the last year of life

#### All patients (*N* = 33,359)

The results of the linear regressions indicated that outpatient PC in the last year of life was associated with significantly higher total healthcare costs (3,523€, 95% CI[3,091, 3,955], *p* < 0.001). The models for costs associated with particular healthcare sectors showed similar results. Specifically, outpatient PC was associated with significantly higher outpatient physician costs (452€, 95% CI[364, 539], *p* < 0.001) and pharmaceutical costs (1,757€, 95% CI[1,551, 1,964], *p* < 0.001). However, no significant difference in hospital costs emerged between patients who did and did not receive outpatient PC (44€, 95% CI[-310, 399], *p* = 0.808). Detailed information on the multivariate models predicting costs are presented in the Appendix (see Additional file 1 for Additional Tables 1–4).

#### Patients with cancer (*n* = 11,173)

The models showed that, for patients with cancer, outpatient PC in the last year of life was associated with higher total healthcare costs (5,567€, 95% CI[4,727, 6,407],* p* < 0.001), outpatient physician costs (592€, 95% CI[448, 737], *p* < 0.001) and pharmaceutical costs (3,334€, 95% CI[2,808, 3,861], p < 0.001), but lower hospital costs (-580€, 95% CI[-1,213, 53], *p* = 0.072). Detailed results of the regression models for cancer patients are shown in the Appendix (see Additional file 2 for Additional tables 5–8).

#### Patients with non-cancer diseases (*n* = 22,186)

The models indicated that, for patients with non-cancer diseases, outpatient PC was associated with significantly higher total healthcare costs (1,512€, 95% CI[1,039, 1,985], *p* < 0.001), but similar hospital costs (301€, 95% CI[-125, 728], *p* = 0.166), relative to patients with non-cancer diseases who did not receive outpatient PC. Additionally, the models showed significantly higher outpatient physician costs (323€, 95% CI[212, 434], *p* < 0.001) and pharmaceutical costs for non-cancer patients receiving outpatient PC (385€, 95% CI [274, 496], *p* < 0,0.001). Detailed results of the regression models for non-cancer patients are presented in the Appendix (see Additional file 3 for Additional tables 9–12).

## Discussion

The current analysis aimed at assessing the impact of outpatient PC on healthcare expenditures in the last year of life, in the context of the German healthcare system. Moreover, a secondary aim was to assess differences in end-of-life costs between cancer and non-cancer patients who were and were not receiving outpatient PC.

### Main results

The descriptive results showed average costs amounting to 21,229€ in the last year of life, per patient, with higher costs associated with cancer patients (30,772€), compared to patients with non-cancer diseases (16,398€). In the group of all patients, those receiving outpatient PC had higher costs in all sectors. Cancer patients receiving outpatient PC had higher total healthcare costs but lower hospital costs. Among non-cancer patients, those receiving outpatient PC had similar total healthcare costs as those who did not receive outpatient PC, but significantly lower hospital costs. Although outpatient PC was associated with higher hospital costs in the group of all patients, this finding was not borne out in both subgroups. At first, this result may appear contradictory. Nevertheless, it is an example of the well-known phenomenon of Simpson’s paradox, by which a group trend reverses when different variables are combined [24]. The results of the regression models showed significantly higher total healthcare costs, outpatient physician costs and pharmaceutical costs in patients receiving outpatient PC for the total cohort, as well as for the two subgroups (i.e. cancer patients and non-cancer patients). Additionally, in both the total cohort and the two subgroups, hospital costs did not differ significantly between patients receiving outpatient PC and patients who did not receive outpatient PC. In cancer patients, lower hospital costs were registered by patients receiving outpatient PC, but the difference did not pass the threshold for significance (*p* = 0.072).

### The impact of outpatient PC on healthcare expenditure in the last year of life

Our findings do not support the hypothesis of cost savings through the provision of PC. This corresponds with two Canadian studies that show PC is often provided on top of routine care and that it leads to additional costs [25, 26]. In this analysis, it remains unclear which services were responsible for higher costs and whether they were necessary or not. Of note, cost reduction is not a primary goal of PC. Rather, PC is designed to improve quality of life in the last phase of life, based on (for example) good symptom control, independent of the associated costs. International studies have found that associations between PC and lower healthcare costs have been predominantly due to reduced hospital admissions, shorter hospital stays and the avoidance of aggressive treatments [11, 27].

The present results align with the German study by Gaertner et al. [18]. Although Gaertner et al. focused on inpatient PC and used a different methodological approach. Their study found no reduction in healthcare costs associated with inpatient PC.

Regarding hospital costs, for the total sample and for non-cancer patients, no significant cost differences were found, based on the regression models. Nevertheless, it is important to note that financing systems and access to healthcare services are not comparable internationally. For example, annual health expenditure per capita in the USA is twice as high as in Germany. In addition, total hospital expenditures per capita in particular are much more expensive in the USA (2,634 USD) than in Germany (1,245 USD, adjusted for purchasing power parity) [28]. As a result, it is possible that cost savings in the USA may be due to the higher impact of avoided hospital stays.

The results of the regression model showed slightly lower hospital costs for cancer patients receiving outpatient PC. This finding is in line with the objective of PC to prevent (unwanted) hospital stays and to reduce lengths of hospital stays [29]. While the difference in costs was not significant, it suggests that outpatient PC is being used more often with cancer patients, while patients with non-cancer diseases may be underserved. In particular, the present results show that more than half of cancer patients received (any type of) outpatient PC, while only a fifth of non-cancer patients received these services.

Another goal of PC is to avoid aggressive therapies (e.g. chemotherapy) in the last phase of life [29]. However, it would be incorrect to assume that chemotherapy is always unnecessary at the end of life. In fact, treatment may be recommended for patients with a fast disease progression, and it may even improve quality of life. The fact that patients receiving outpatient PC had higher pharmaceutical costs may be due to the delivery of medication to alleviate symptoms. This assumption could be supported by the findings of Gaertner et al. that patients with PC received more chemotherapy and opioids [18].

Nonetheless, it is known that overtreatment at the end of life is part of a broader and pervasive challenge of medical service overuse, which is not necessarily in patients’ best interests [30]. The present findings do not address whether the use of resources was due to possible overtreatment, or if outpatient PC had a positive effect on patients’ quality of life. However, previous studies have shown that outpatient PC has beneficial effects in terms of patients’ quality of life and overall satisfaction [31, 32]. For example, most individuals prefer to die in their own home or in an outpatient setting [33], and Willinger et al. showed, in a German study, that the proportion of individuals who died in hospital was lower among those receiving outpatient PC in their last year of life [34]. In particular, the early initiation of outpatient PC has been shown to generate several positive outcomes and to potentially lower costs, due to the avoidance of unnecessary/unwanted treatments [30, 35]. However, a German study based on SHI data showed that outpatient PC tends to be initiated late in the course of treatment, and should be integrated earlier, in practice [36].

### Strengths and limitations

The present retrospective study was based on administrative SHI data, which were originally collected for billing purposes. This dataset enabled the analysis of a large patient cohort with a wide variety of underlying diseases, explicitly taking multi-/comorbidity into account. In addition, the dataset minimised the impact of selection bias, which is often present in studies using primary data. A further strength of the study is that it analysed a variety of costs associated with different healthcare sectors (representing dominant categories of healthcare costs) in the last year of life [7]. However, limitations arose due to gaps in the data, associated with the severity of the underlying diseases, specific symptoms, the main reasons for patients’ outpatient PC and the impact of outpatient PC on patients’ quality of life. Furthermore, while efforts were made to control for various influencing factors, the impact of a specific disease on healthcare costs might significantly differ between individuals, due to illness severity and specific symptoms. For example, treatment costs and the chances of treatment success may considerably differ between patients with different cancer types and patients at different stages of the disease, as well as between patients for whom different treatment options are available. Furthermore, treatment costs are heterogeneous and may vary from a few thousand to several hundred thousand euros [37, 38]. Thus, the present effort to control for morbidity may not have been sufficient. It should also be mentioned that the decision to use PC is voluntary and can be influenced by many factors (for example regional supply structure, attitude of the attending physician to PC or sociodemographic factors) and lead to selection bias [39, 40]. These factors can also influence healthcare expenditure and our model cannot control for all of these variables.

## Conclusions

The present study complements (inter)national research and shows the impact of outpatient PC on healthcare expenditure in the last year of life in Germany. The study also provides insight into different cost sectors. In contrast to the majority of international studies, the present study did not find an association between the provision of outpatient PC and lower healthcare costs. For hospital costs, the results were heterogeneous, though trending towards lower values for cancer patients receiving outpatient PC. Comparability with (inter)national studies remains difficult, because financing systems and access to healthcare services are not comparable internationally. Additional studies should also include different cost sectors in the analyses and thus complement the current research. Furthermore, many studies have recommended the early integration of PC. Further analyses should investigate the connection between the time at which PC is initiated and end-of-life costs.

## Supplementary Information

Additional file 1. The file contains the results of the regression models from all patients (Additional table 1-4).

Additional file 2. The file contains the results of the regression models from patients with cancer (Additional table 5-8).

Additional file 3. The file contains the results of the regression models from patients with non-cancer diseases (Additional table 9-12).

## Data Availability

Data from the statutory health insurance fund AOKN are not publicly available due to data privacy protection regulations.

## Abbreviations

AOKN: Allgemeine Ortskrankenkasse’ Lower Saxony (AOKN) (local statutory health insurance fund)
GPC: Generalist outpatient PC
ICD-10: *International Statistical Classification of Diseases and Related Health Problems* (10Th Revision)
IPC: Intermediate level of PC
OS: Special oncological PC services
PC: Palliative care
SHI: Statutory health insurance
SPC: Specialist outpatient PC
