# Supplementary material for: Impact of outpatient palliative care on healthcare costs in Germany – an analysis of cancer and non-cancer patients based on health insurance data
Source: Health Econ Rev. 2025 Aug 20;15:70. doi: 10.1186/s13561-025-00604-z (PMC12366161; doi:10.1186/s13561-025-00604-z)
Supplement: Supplementary file 1 — Additional file 1. The file contains the results of the regression models from all patients (Additional table 1-4). [file 13561_2025_604_MOESM1_ESM.docx]

***Additional table 1: Total healthcare costs in the last year of life, all patients* (N=*33,359)***

|  |  | **Exp (b)** | **Lower CI** | **Upper CI** | ***SE*** | ***p-*value** |
| --- | --- | --- | --- | --- | --- | --- |
|  | Constant | 12,953.98 | 11,778.60 | 14,129.37 | 599.68 | <0.001 |
| **Sex (female, reference: male)** | Sex | -768.95 | -1,148.19 | -389.70 | 193.49 | <0.001 |
| **Age at death**  **(years)**  **Reference: 0-49** | 50-69 | -3,965.69 | -5,205.37 | -2,726.00 | 632.48 | <0.001 |
|  | 70-79 | -9,384.98 | -10,648.79 | -8,121.17 | 644.79 | <0.001 |
|  | 80-89 | -16,655.28 | -17,913.65 | -15,396.91 | 642.02 | <0.001 |
|  | older than 89 | -22,424.59 | -23,725.41 | -21,123.77 | 663.67 | <0.001 |
| **Care degree**  **(reference: no care degree)** | 1 | 5,697.44 | 4,181.33 | 7,213.55 | 773.51 | <0.001 |
|  | 2 | 7,489.91 | 6,846.46 | 8,133.37 | 328.29 | <0.001 |
|  | 3 | 8,730.99 | 8,099.43 | 9,362.54 | 322.22 | <0.001 |
|  | 4 | 8,825.16 | 8,198.37 | 9,451.96 | 319.79 | <0.001 |
|  | 5 | 8,199.71 | 7,524.23 | 8,875.20 | 344.63 | <0.001 |
| **Morbidity** | Dementia | -2,811.65 | -3,247.15 | -2,376.15 | 222.19 | <0.001 |
|  | Diabetes | 791.38 | 415.03 | 1,167.73 | 192.01 | <0.001 |
|  | Hypertension | 2,855.35 | 2,325.47 | 3,385.23 | 270.34 | <0.001 |
|  | Coronary heart disease | 1,215.07 | 808.92 | 1,621.21 | 207.21 | <0.001 |
|  | Heart failure | 4,056.86 | 3,652.59 | 4,461.13 | 206.26 | <0.001 |
|  | Asthma | 692.69 | -42.35 | 1,427.72 | 375.01 | 0.065 |
|  | COPD | 930.72 | 494.47 | 1,366.97 | 222.57 | <0.001 |
|  | Depressive disorder | 1,714.30 | 1,307.62 | 2,120.97 | 207.48 | <0.001 |
|  | Renal failure | 6,701.54 | 6,314.94 | 7,088.14 | 197.24 | <0.001 |
|  | Solid tumors | 3,734.93 | 3,229.72 | 4,240.14 | 257.76 | <0.001 |
|  | Metastatic cancer | 10,730.50 | 10,079.33 | 11,381.67 | 332.22 | <0.001 |
|  | Lymphoid/haematopoietic cancer | 11,470.49 | 10,512.40 | 12,428.58 | 488.81 | <0.001 |
|  | Parkinson‘s | -605.51 | -1,394.93 | 183.92 | 402.76 | 0.133 |
|  | Myocardial infarction | 5,210.82 | 4,404.84 | 6,016.80 | 411.21 | <0.001 |
|  | Stroke | 9,137.40 | 8,474.11 | 9,800.69 | 338.41 | <0.001 |
| **Receiving outpatient PC** | Outpatient PC (yes/no) | 3,523.09 | 3,091.49 | 3,954.70 | 220.20 | <0.001 |

***Additional table 2: Hospital costs in the last year of life, all patients* (N=*33,359)***

|  |  | **Exp (b)** | **Lower CI** | **Upper CI** | ***SE*** | ***p*-value** |
| --- | --- | --- | --- | --- | --- | --- |
|  | Constant | 9,208.99 | 8,243.52 | 10,174.45 | 492.58 | <0.001 |
| **Sex (female, reference: male)** | Sex | -665.87 | -977.39 | -354.36 | 158.93 | <0.001 |
| **Age at death**  **(years)**  **Reference: 0-49** | 50-69 | -2,921.27 | -3,939.56 | -1,902.99 | 519.52 | <0.001 |
|  | 70-79 | -6,197.23 | -7,235.33 | -5,159.13 | 529.63 | <0.001 |
|  | 80-89 | -11,259.94 | -12,293.57 | -10,226.31 | 527.35 | <0.001 |
|  | older than 89 | -15,532.29 | -16,600.79 | -14,463.78 | 545.14 | <0.001 |
| **Care degree**  **(reference: no care degree)** | 1 | 3,826.86 | 2,581.52 | 5,072.19 | 635.36 | <0.001 |
|  | 2 | 4,401.40 | 3,872.86 | 4,929.94 | 269.66 | <0.001 |
|  | 3 | 4,420.98 | 3,902.22 | 4,939.74 | 264.67 | <0.001 |
|  | 4 | 4,332.10 | 3,817.25 | 4,846.95 | 262.67 | <0.001 |
|  | 5 | 4,171.60 | 3,616.75 | 4,726.44 | 283.08 | <0.001 |
| **Morbidity** | Dementia | -1,103.48 | -1,461.20 | -745.76 | 182.51 | <0.001 |
|  | Diabetes | 267.37 | -41.76 | 576.51 | 157.72 | <0.090 |
|  | Hypertension | 2,018.65 | 1,583.41 | 2,453.89 | 222.06 | <0.001 |
|  | Coronary heart disease | 865.11 | 531.51 | 1,198.72 | 170.20 | <0.001 |
|  | Heart failure | 4,003.06 | 3,670.99 | 4,335.13 | 169.42 | <0.001 |
|  | Asthma | 421.36 | -182.41 | 1,025.12 | 308.04 | 0.171 |
|  | COPD | 954.88 | 596.54 | 1,313.21 | 182.82 | <0.001 |
|  | Depressive disorder | 1,354.93 | 1,020.88 | 1,688.97 | 170.43 | <0.001 |
|  | Renal failure | 5,744.57 | 5,427.02 | 6,062.13 | 162.02 | <0.001 |
|  | Solid tumors | 2,059.06 | 1,644.07 | 2,474.04 | 211.72 | <0.001 |
|  | Metastatic cancer | 3,581.19 | 3,046.32 | 4,116.06 | 272.89 | <0.001 |
|  | Lymphoid/haematopoietic cancer | 5,816.73 | 5,029.75 | 6,603.71 | 401.51 | <0.001 |
|  | Parkinson‘s | -464.53 | -1,112.97 | 183.91 | 330.83 | 0.160 |
|  | Myocardial infarction | 5,451.69 | 4,789.65 | 6,113.72 | 337.77 | <0.001 |
|  | Stroke | 9,835.35 | 9,290.52 | 10,380.18 | 277.97 | <0.001 |
| **Receiving outpatient PC** | Outpatient PC (yes/no) | 44.03 | -310.49 | 398.55 | 180.87 | 0.808 |

***Additional table 3: Outpatient physician costs in the last year of life, all patients* (N=*33,359)***

|  |  | **Exp (b)** | **Lower CI** | **Upper CI** | ***SE*** | ***p*-value** |
| --- | --- | --- | --- | --- | --- | --- |
|  | Constant | 738.64 | 500.69 | 976.59 | 121.40 | <0.001 |
| **Sex (female, reference: male)** | Sex | -116.87 | -193.64 | -40.09 | 39.17 | 0.003 |
| **Age at death**  **(years)**  **Reference: 0-49** | 50-69 | -29.23 | -280.20 | 221.74 | 128.04 | 0.819 |
|  | 70-79 | -371.05 | -626.90 | -115.20 | 130.53 | 0.004 |
|  | 80-89 | -772.82 | -1,027.57 | -518.07 | 129.97 | <0.001 |
|  | older than 89 | -1,179.27 | -1,442.61 | -915.92 | 134.36 | <0.001 |
| **Care degree**  **(reference: no care degree)** | 1 | 390.76 | 83.84 | 697.69 | 156.59 | 0.013 |
|  | 2 | 716.35 | 586.09 | 846.61 | 66.46 | <0.001 |
|  | 3 | 971.74 | 843.89 | 1,099.60 | 65.23 | <0.001 |
|  | 4 | 885.35 | 758.46 | 1,012.24 | 64.74 | <0.001 |
|  | 5 | 787.98 | 651.24 | 924.73 | 69.77 | <0.001 |
| **Morbidity** | Dementia | -269.84 | -358.00 | -181.67 | 44.98 | <0.001 |
|  | Diabetes | 225.66 | 149.47 | 301.86 | 38.87 | <0.001 |
|  | Hypertension | 325.73 | 218.46 | 433.01 | 54.73 | <0.001 |
|  | Coronary heart disease | 303.89 | 221.67 | 386.11 | 41.95 | <0.001 |
|  | Heart failure | 130.28 | 48.44 | 212.12 | 41.76 | 0.002 |
|  | Asthma | -69.89 | -218.70 | 78.91 | 75.92 | 0.357 |
|  | COPD | 51.53 | -36.78 | 139.85 | 45.06 | 0.253 |
|  | Depressive disorder | 42.42 | -39.90 | 124.75 | 42.00 | 0.312 |
|  | Renal failure | 681.81 | 603.55 | 760.08 | 39.93 | <0.001 |
|  | Solid tumors | 348.41 | 246.14 | 450.69 | 52.18 | <0.001 |
|  | Metastatic cancer | 584.05 | 452.22 | 715.87 | 67.26 | <0.001 |
|  | Lymphoid/haematopoietic cancer | 815.75 | 621.79 | 1,009.71 | 98.96 | <0.001 |
|  | Parkinson‘s | -86.62 | -246.43 | 73.20 | 81.54 | 0.288 |
|  | Myocardial infarction | -95.16 | -258.32 | 68.01 | 83.25 | 0.253 |
|  | Stroke | -108.06 | -242.34 | 26.22 | 68.51 | 0.115 |
| **Receiving outpatient PC** | Outpatient PC (yes/no) | 451.74 | 364.36 | 539.11 | 44.58 | <0.001 |

***Additional table 4: Pharmaceutical costs in the last year of life, all patients* (N=*33,359)***

|  |  | **Exp (b)** | **Lower CI** | **Upper CI** | ***SE*** | ***p*-value** |
| --- | --- | --- | --- | --- | --- | --- |
|  | Constant | 2,729.67 | 2,167.71 | 3,291.63 | 286.71 | <0.001 |
| **Sex (female, reference: male)** | Sex | -155.25 | -336.57 | 26.07 | 92.51 | 0.093 |
| **Age at death**  **(years)**  **Reference: 0-49** | 50-69 | -688.83 | -1,281.54 | -96.13 | 302.39 | 0.023 |
|  | 70-79 | -2,206.48 | -2,810.72 | -1,602.24 | 308.28 | <0.001 |
|  | 80-89 | -3,807.19 | -4,408.83 | -3,205.56 | 306.95 | <0.001 |
|  | older than 89 | -4,660.44 | -5,282.37 | -4,038.51 | 317.31 | <0.001 |
| **Care degree**  **(reference: no care degree)** | 1 | 1,242.65 | 517.79 | 1,967.51 | 369.82 | <0.001 |
|  | 2 | 2,093.31 | 1,785.67 | 2,400.95 | 156.96 | <0.001 |
|  | 3 | 2,807.10 | 2,505.15 | 3,109.05 | 154.05 | <0.001 |
|  | 4 | 2,817.40 | 2,517.72 | 3,117.07 | 152.89 | <0.001 |
|  | 5 | 2,618.15 | 2,295.19 | 2,941.10 | 164.77 | <0.001 |
| **Morbidity** | Dementia | -1,038.02 | -1,246.24 | -829.81 | 106.23 | <0.001 |
|  | Diabetes | 315.07 | 135.13 | 495.00 | 91.80 | 0.001 |
|  | Hypertension | 493.59 | 240.25 | 746.93 | 129.25 | <0.001 |
|  | Coronary heart disease | -21.89 | -216.07 | 172.29 | 99.07 | 0.825 |
|  | Heart failure | 16.22 | -177.07 | 209.50 | 98.61 | 0.869 |
|  | Asthma | 286.30 | -65.12 | 637.73 | 179.30 | 0.110 |
|  | COPD | -39.33 | -247.91 | 169.24 | 106.41 | 0.712 |
|  | Depressive disorder | 223.06 | 28.62 | 417.49 | 99.20 | 0.025 |
|  | Renal failure | 416.88 | 232.04 | 601.72 | 94.30 | <0.001 |
|  | Solid tumors | 868.26 | 626.72 | 1,109.81 | 123.24 | <0.001 |
|  | Metastatic cancer | 5,757.32 | 5,445.99 | 6,068.64 | 158.84 | <0.001 |
|  | Lymphoid/haematopoietic cancer | 4,642.42 | 4,184.35 | 5,100.49 | 233.70 | <0.001 |
|  | Parkinson‘s | 99.56 | -277.86 | 476.99 | 192.56 | 0.605 |
|  | Myocardial infarction | -130.40 | -515.75 | 254.94 | 196.60 | 0.507 |
|  | Stroke | -470.12 | -787.24 | -152.99 | 161.79 | 0.004 |
| **Receiving outpatient PC** | Outpatient PC (yes/no) | 1,757.33 | 1,550.98 | 1,963.68 | 105.28 | <0.001 |

Additional file 1 contains the results of the regression models from all patients for total healthcare costs (Additional table 1), hospital costs (Additional table 2), outpatient physician costs (Additional table 3) and pharmaceutical costs (Additional table 4).
