# Supplementary material for: Impact of outpatient palliative care on healthcare costs in Germany – an analysis of cancer and non-cancer patients based on health insurance data
Source: Health Econ Rev. 2025 Aug 20;15:70. doi: 10.1186/s13561-025-00604-z (PMC12366161; doi:10.1186/s13561-025-00604-z)
Supplement: Supplementary file 3 — Additional file 3. The file contains the results of the regression models from patients with non-cancer diseases (Additional table 9-12). [file 13561_2025_604_MOESM3_ESM.docx]

***Additional table 9: Total healthcare costs in the last year of life, patients with non-cancer diseases (*n*=22,186)***

|  |  | **Exp (b)** | **Lower CI** | **Upper CI** | ***SE*** | ***p*-value** |
| --- | --- | --- | --- | --- | --- | --- |
|  | Constant | 8,532.75 | 7,389.35 | 9,676.16 | 583.35 | <0.001 |
| **Sex (female, reference: male)** | Sex | -826.73 | -1,229.19 | -424.27 | 205.33 | <0.001 |
| **Age at death**  **(years)**  **Reference: 0-49** | 50-69 | -2,938.93 | -4,180.65 | -1,697.21 | 633.51 | <0.001 |
|  | 70-79 | -5,908.28 | -7,181.71 | -4,634.85 | 649.69 | <0.001 |
|  | 80-89 | -12,084.10 | -13,345.45 | -10,822.74 | 643.53 | <0.001 |
|  | older than 89 | -17,422.66 | -18,722.80 | -16,122.51 | 663.32 | <0.001 |
| **Care degree**  **(reference: no care degree)** | 1 | 5,479.70 | 3,946.30 | 7,013.10 | 782.32 | <0.001 |
|  | 2 | 6,395.30 | 5,715.53 | 7,075.07 | 346.81 | <0.001 |
|  | 3 | 7,366.04 | 6,703.31 | 8,028.76 | 338.11 | <0.001 |
|  | 4 | 7,670.10 | 7,004.62 | 8,335.57 | 339.52 | <0.001 |
|  | 5 | 7,444.96 | 6,745.85 | 8,144.08 | 356.68 | <0.001 |
| **Morbidity** | Dementia | -1,926.18 | -2,367.93 | -1,484.44 | 225.37 | <0.001 |
|  | Diabetes | 1,081.29 | 690.28 | 1,472.31 | 199.49 | <0.001 |
|  | Hypertension | 3,250.95 | 2,696.12 | 3,805.78 | 283.06 | <0.001 |
|  | Coronary heart disease | 1,636.66 | 1,219.20 | 2,054.13 | 212.98 | <0.001 |
|  | Heart failure | 4,513.73 | 4,094.91 | 4,932.54 | 213.67 | <0.001 |
|  | Asthma | 167.15 | -615.24 | 949.54 | 399.16 | 0.675 |
|  | COPD | 2,108.18 | 1,644.12 | 2,572.24 | 236.76 | <0.001 |
|  | Depressive disorder | 1,453.20 | 1,030.65 | 1,875.74 | 215.58 | <0.001 |
|  | Renal failure | 7,427.37 | 7,020.08 | 7,834.65 | 207.79 | <0.001 |
|  | Parkinson‘s | -326.42 | -1,088.62 | 435.77 | 388.86 | 0.401 |
|  | Myocardial infarction | 5,194.25 | 4,417.62 | 5,970.88 | 396.23 | <0.001 |
|  | Stroke | 9,855.22 | 9,198.69 | 10,511.75 | 334.95 | <0.001 |
| **Receiving outpatient PC** | Outpatient PC (yes/no) | 1,511.84 | 1,038.90 | 1,984.78 | 241.29 | <0.001 |

***Additional table 10: Hospital costs costs in the last year of life, patients with non-cancer diseases (*n*=22,186)***

|  |  | **Exp (b)** | **Lower CI** | **Upper CI** | ***SE*** | ***p*-value** |
| --- | --- | --- | --- | --- | --- | --- |
|  | Constant | 6,841.00 | 5,810.08 | 7,871.92 | 525.96 | <0.001 |
| **Sex (female, reference: male)** | Sex | -790.43 | -1,153.30 | -427.57 | 185.13 | <0.001 |
| **Age at death**  **(years)**  **Reference: 0-49** | 50-69 | -2,373.20 | -3,492.76 | -1,253.64 | 571.19 | <0.001 |
|  | 70-79 | -4,646.07 | -5,794.22 | -3,497.91 | 585.77 | <0.001 |
|  | 80-89 | -9,802.69 | -10,939.96 | -8,665.42 | 580.22 | <0.001 |
|  | older than 89 | -13,943.58 | -15,115.83 | -12,771.34 | 598.06 | <0.001 |
| **Care degree**  **(reference: no care degree)** | 1 | 4,305.50 | 2,922.95 | 5,688.05 | 705.36 | <0.001 |
|  | 2 | 4,346.57 | 3,733.67 | 4,959.47 | 312.69 | <0.001 |
|  | 3 | 4,622.04 | 4,024.51 | 5,219.57 | 304.85 | <0.001 |
|  | 4 | 4,806.67 | 4,206.67 | 5,406.68 | 306.12 | <0.001 |
|  | 5 | 4,338.94 | 3,708.60 | 4,969.28 | 321.59 | <0.001 |
| **Morbidity** | Dementia | -896.01 | -1,294.30 | -497.73 | 203.20 | <0.001 |
|  | Diabetes | 284.32 | -68.22 | 636.87 | 179.86 | 0.114 |
|  | Hypertension | 2,375.73 | 1,875.48 | 2,875.97 | 255.22 | <0.001 |
|  | Coronary heart disease | 1,113.64 | 737.25 | 1,490.04 | 192.03 | <0.001 |
|  | Heart failure | 4,098.77 | 3,721.15 | 4,476.38 | 192.65 | <0.001 |
|  | Asthma | 105.71 | -599.71 | 811.13 | 359.89 | 0.769 |
|  | COPD | 1,593.31 | 1,174.90 | 2,011.72 | 213.47 | <0.001 |
|  | Depressive disorder | 1,263.13 | 882.15 | 1,644.11 | 194.37 | <0.001 |
|  | Renal failure | 6,014.74 | 5,647.53 | 6,381.96 | 187.35 | <0.001 |
|  | Parkinson‘s | -668.43 | -1,355.64 | 18.79 | 350.61 | 0.057 |
|  | Myocardial infarction | 5,650.76 | 4,950.54 | 6,350.99 | 357.25 | <0.001 |
|  | Stroke | 10,412.75 | 9,820.80 | 11,004.69 | 302.00 | <0.001 |
| **Receiving outpatient PC** | Outpatient PC (yes/no) | 301.47 | -124.94 | 727.88 | 217.55 | 0.166 |

***Additional table 11: Outpatient physician costs in the last year of life, patients with non-cancer diseases (*n*=22,186)***

|  |  | **Exp (b)** | **Lower CI** | **Upper CI** | ***SE*** | ***p*-value** |
| --- | --- | --- | --- | --- | --- | --- |
|  | Constant | 487.24 | 219.40 | 755.08 | 136.65 | <0.001 |
| **Sex (female, reference: male)** | Sex | -89.04 | -183.32 | 5.23 | 48.10 | 0.064 |
| **Age at death**  **(years)**  **Reference: 0-49** | 50-69 | -86.82 | -377.68 | 204.05 | 148.40 | 0.559 |
|  | 70-79 | -279.46 | -577.75 | 18.84 | 152.19 | 0.066 |
|  | 80-89 | -536.80 | -832.27 | -241.34 | 150.74 | <0.001 |
|  | older than 89 | -961.98 | -1,266.53 | -657.43 | 155.38 | <0.001 |
| **Care degree**  **(reference: no care degree)** | 1 | 448.74 | 89.55 | 807.93 | 183.25 | 0.014 |
|  | 2 | 669.96 | 510.73 | 829.20 | 81.24 | <0.001 |
|  | 3 | 886.36 | 731.12 | 1,041.60 | 79.20 | <0.001 |
|  | 4 | 846.43 | 690.55 | 1,002.32 | 79.53 | <0.001 |
|  | 5 | 782.82 | 619.06 | 946.59 | 83.55 | <0.001 |
| **Morbidity** | Dementia | -215.90 | -319.38 | -112.43 | 52.79 | <0.001 |
|  | Diabetes | 234.82 | 143.23 | 326.41 | 46.73 | <0.001 |
|  | Hypertension | 393.07 | 263.11 | 523.04 | 66.31 | <0.001 |
|  | Coronary heart disease | 324.34 | 226.55 | 422.13 | 49.89 | <0.001 |
|  | Heart failure | 135.47 | 37.37 | 233.58 | 50.05 | 0.007 |
|  | Asthma | -145.47 | -328.74 | 37.80 | 93.50 | 0.120 |
|  | COPD | 97.01 | -11.69 | 205.72 | 55.46 | 0.080 |
|  | Depressive disorder | -13.80 | -112.78 | 85.18 | 50.50 | 0.785 |
|  | Renal failure | 795.84 | 700.44 | 891.25 | 48.67 | <0.001 |
|  | Parkinson‘s | -17.97 | -196.51 | 160.57 | 91.09 | 0.844 |
|  | Myocardial infarction | -155.94 | -337.86 | 25.98 | 92.81 | 0.093 |
|  | Stroke | -171.56 | -325.34 | -17.77 | 78.46 | 0.029 |
| **Receiving outpatient PC** | Outpatient PC (yes/no) | 322.82 | 212.04 | 433.60 | 56.52 | <0.001 |

***Additional table 12: Pharmaceutical costs in the last year of life, patients with non-cancer diseases (*n*=22,186)***

|  |  | **Exp (b)** | **Lower CI** | **Upper CI** | ***SE*** | ***p*-value** |
| --- | --- | --- | --- | --- | --- | --- |
|  | Constant | 1,135.95 | 867.42 | 1,404.48 | 137.00 | <0.001 |
| **Sex (female, reference: male)** | Sex | 55.73 | -38.79 | 150.25 | 48.22 | 0.248 |
| **Age at death**  **(years)**  **Reference: 0-49** | 50-69 | -423.64 | -715.26 | -132.02 | 148.78 | 0.004 |
|  | 70-79 | -865.94 | -1,165.01 | -566.87 | 152.58 | <0.001 |
|  | 80-89 | -1,603.64 | -1,899.88 | -1,307.41 | 151.13 | <0.001 |
|  | older than 89 | -2,307.09 | -2,612.43 | -2,001.75 | 155.78 | <0.001 |
| **Care degree**  **(reference: no care degree)** | 1 | 722.85 | 362.73 | 1,082.98 | 183.73 | <0.001 |
|  | 2 | 1,327.46 | 1,167.81 | 1,487.10 | 81.45 | <0.001 |
|  | 3 | 1,762.06 | 1,606.42 | 1,917.71 | 79.41 | <0.001 |
|  | 4 | 1,869.63 | 1,713.34 | 2,025.91 | 79.74 | <0.001 |
|  | 5 | 2,146.64 | 1,982.45 | 2,310.83 | 83.77 | <0.001 |
| **Morbidity** | Dementia | -689.18 | -792.92 | -585.43 | 52.93 | <0.001 |
|  | Diabetes | 549.35 | 457.52 | 641.18 | 46.85 | <0.001 |
|  | Hypertension | 473.96 | 343.66 | 604.26 | 66.48 | <0.001 |
|  | Coronary heart disease | 170.96 | 72.92 | 269.01 | 50.02 | <0.001 |
|  | Heart failure | 293.33 | 194.97 | 391.69 | 50.18 | <0.001 |
|  | Asthma | 183.10 | -0.65 | 366.85 | 93.74 | 0.051 |
|  | COPD | 355.90 | 246.92 | 464.89 | 55.60 | <0.001 |
|  | Depressive disorder | 184.19 | 84.95 | 283.43 | 50.63 | <0.001 |
|  | Renal failure | 620.22 | 524.56 | 715.87 | 48.80 | <0.001 |
|  | Parkinson‘s | 434.47 | 255.46 | 613.47 | 91.33 | <0.001 |
|  | Myocardial infarction | -295.64 | -478.03 | -113.24 | 93.05 | 0.001 |
|  | Stroke | -357.97 | -512.16 | -203.78 | 78.66 | <0.001 |
| **Receiving outpatient PC** | Outpatient PC (yes/no) | 384.81 | 273.74 | 495.88 | 56.67 | <0.001 |

Additional file 3 contains the results of the regression models from patients with non-cancer diseases for total healthcare costs (Additional table 9), hospital costs (Additional table 10), outpatient physician costs (Additional table 11) and pharmaceutical costs (Additional table 12).
